# Integrating terminologies into standard SQL: a new approach for research on routine data

**DOI:** 10.1186/s13326-019-0199-z

**Published:** 2019-04-24

**Authors:** André Sander, Roland Wauer

**Affiliations:** 1 ID GmbH & Co. KGaA, Platz vor dem Neuen Tor 2, 10115 Berlin, Germany; 2 Klinik für Neonatologie, Charité-Universitätsmedizin Berlin, 10098 Berlin, Germany

**Keywords:** Ontology-based queries, Terminology server, Translational research, Infant mortality, Data mining (knowledge discovery)

## Abstract

**Background:**

Most electronic medical records still contain large amounts of free-text data. Semantic evaluation of such data requires the data to be encoded with sufficient classifications or transformed into a knowledge-based database.

**Methods:**

We present an approach that allows databases accessible via SQL (Structured Query Language) to be searched directly through semantic queries without the need for further transformations. Therefore, we developed I) an extension to SQL named Ontology-SQL (O-SQL) that allows to use semantic expressions, II) a framework that uses a standard terminology server to annotate free-text containing database tables and III) a parser that rewrites O-SQL to SQL, so that such queries can be passed to the database server.

**Results:**

I) We compared several semantic queries published to date and were able to reproduce them in a reduced, highly condensed form. II) The quality of the annotation process was measured against manual annotation, and we found a sensitivity of 97.62% and a specificity of 100.00%. III) Different semantic queries were analyzed, and measured with F-scores between 0.91 and 0.98.

**Conclusions:**

We showed that systematic analysis of free-text-containing medical records is possible with standard tools. The seamless connection of ontologies and standard technologies from the database field represents an important constituent of unstructured data analysis. The developed technology can be readily applied to relationally organized data and supports the increasingly important field of translational research.

**Electronic supplementary material:**

The online version of this article (10.1186/s13326-019-0199-z) contains supplementary material, which is available to authorized users.

## Background

The launching of a working group on the “use of electronic medical records for clinical research” [1] in 2011 by the GMDS (Deutsche Gesellschaft für Medizinische Informatik, Biometrie und Epidemiologie) is evidence of the enormous importance of medical records for research; however, it also underlines the difficulties that arise when trying to analyze these data. In the following, we therefore explain an approach that addresses the efficient usage of medical records in well-established structures. We introduce an approach that integrates free-text-based query terms into standard SQL and thus allows such queries to be run on existing database systems. The free text is mapped to a terminology and semantically interpreted using an ontology provided by a terminology server.

## Related work

Two major problems are addressed with our approach: I) the semantic structuring and evaluation of free text and II) the querying of such information from medical records.

Many research papers have presented different approaches to the semantic structuring of free text. The outcomes vary in many aspects; however, in general, these approaches provide good to excellent results. Recent NLP (natural language processing)-based mapping systems were published by Friedman et al. in 2004 [2] and later by Savova et al. [3], both of which exhibited high accuracy. Elkin et al. analyzed mapping algorithms with the SNOMED (systematized nomenclature of medicine) ontology on chest X-ray reports with excellent results [4]. A similar task for pathology was recently presented by Allones et al. but required further improvement [5]. In the German language, a 2015 paper presented by Toepfer et al. showed very good results [6]. Some well-established mapping tools have been used by many groups and were evaluated in [7, 8]. One of the core challenges of these tools is the disambiguation of mapping alternatives, which was currently achieved best by Zwicklbauer et al., who developed DoSeR [9].

Many attempts to enable knowledge-based querying of information from medical records have been described; for example, Hogarth et al. suggested the so-called TQL (terminology query language), which encompassed SQL’s idea of universality [10]. SPARQL (SPARQL Protocol and RDF Query Language) in particular has established itself in many areas as the de facto standard [11]. However, for specific problems, such as mapping between ontologies, additional internal query languages have been developed, even in recent years [12]. There has been an evolution of integrated frameworks for the implementation of browser-based knowledge systems since early on [13, 14]. Today, SPARQL- and OWL (Web Ontology Language)-based systems are successfully implemented for defined applications, such as the management of blood pressure or hypertension [15]. In the area of infectious diseases, Kama et al. used the concept of “a semantic data warehouse” that integrates OLAP (online analytical processing) techniques [16]. Nevertheless, in this approach, specific query languages have remained unaltered in their respective domains. Epstein et al. therefore chose to implement and integrate needed subsystems (e.g., NLP pipelines) into SQL [17]. One of the most recent and interesting approaches came from Zheng et al., who have extended standard SQL with “semantic constructs” [18]. Nevertheless, for this purpose, numerous algorithms have been implemented in the middleware instead of consequently outsourcing them to a terminology server.

Recently, the field of OBDA (ontology-based data access) has been examined as a profound theoretical basis. However, these approaches are currently limited to specific types of databases and their querying languages [19].

Classification-based approaches (e.g., ICD (International Classification of Diseases)-encoded data) are also used in current attempts to integrate heterogeneous data sources for cohort formation [20]. This approach, however, is associated with three major problems: I) loss of specificity [21], II) low interoperability [22] and III) low stability over time [22].

We present an approach that overcomes many of the described technical hurdles and still achieves comparable or even better results.

## Materials

In the work presented here, we use epicrises from 1868 patients collected from free-text medical records. These data were captured in the period from 1973 to 1989 in former East Berlin/East Germany by the Commission on the Reduction of the Infant Mortality Rate^1^ [23]. The medical records of all newborns who died in the first 28 days of life were analyzed, discussed and evaluated by this Commission and classified based on their avoidability. Avoidability was categorized from a medical and social point of view into “non-avoidable”, “conditionally avoidable” and “avoidable”. Subsequently, measures were taken in different areas, ranging from staff training to structural changes, such as the centralized treatment of certain risk groups.

**Fig. 1:**
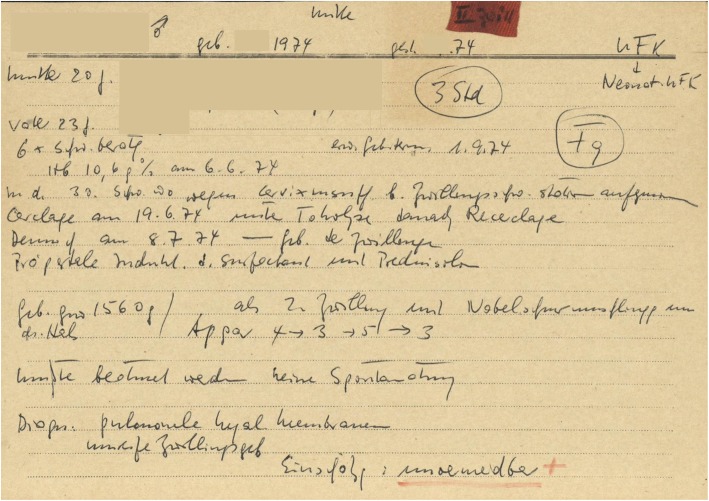
Original sample of a handwritten index card (anonymized). In preparation for each commission meeting on reducing infant mortality (IM), the chairman of the commission prepared such an index card (*N* = 1868). The index cards contain basic demographic data regarding the mother and child and free text regarding anamnesis, birth, postnatal treatment and course of death. The index cards also contain a color-coding system denoting a premature birth (a red mark on top of the card) and a (lethal) malformation (a yellow mark). Additionally, the index cards included the final judgment made by the commission if the case was avoidable, conditionally avoidable or not avoidable. In many cases, the index cards also contained a revision of the initial judgment and an explanatory statement

At that time, data from the original medical records (pregnancy card, birth history logs, all hospital documents, reports of hospital stay, reports of interventions and so on) were stored on handwritten index cards in DIN A5 (see Fig. 1). Apart from some structured properties, these data mainly included clinical text – as defined in [24] – addressing anamnesis, course of birth, treatment and postmortem classification. In each case, both peri- and postnatal care were described in detail, and a differentiated assessment of the cause of death was carried out. When creating the index cards, color highlighting was used to classify cases into the categories “premature birth” (red highlighting) or “lethal malformation” (yellow highlighting) (see Fig. 1 which has a red highlighting).

Digitalization of the cards was performed in two steps: first, a professional service provider for archiving paper-based patient records scanned the index cards and provided high-resolution images of the front and back sides of the cards. In the second step, the cards were manually transcribed into an SQL database. Spelling and grammar, however, were copied exactly. Quality assurance was implemented by a three-stage release process (involving three independent transcriptionists). In the final step, all values of the structured data items were analyzed by A-Z analysis for implausible data.

## Methods

The central idea of the approach presented here consists of integrating a terminology server into an SQL-based RDBMS (Relational Database Management System) and extending the SQL language itself by adding the ability to formulate semantic criteria within the query with free text. Hence, the approach comprises two components:

I) Semantic structuring and annotation of the RDBMS tables, and
II) Syntactic extension of standard SQL (“Ontology-SQL”).

### Semantic structuring and annotation

We chose to store the semantic annotation by using additional tables to extend the database schema instead of changing existing tables. This approach offers the advantage that the data tables that store the annotations can be created in their own logical instance of an existing database server.

**Fig. 2:**
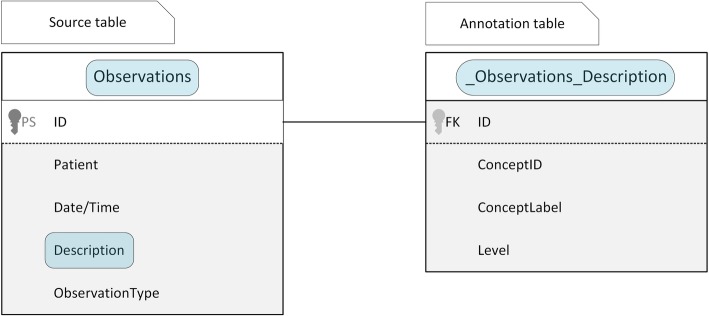
For each table (“aTableName”) and each column (“aColumnName_x”) enabled for semantic queries, an annotation table is created with the naming scheme “[_][aTableName][_][aColumnName_x]”. The annotation table is linked to the source table via the unique primary key. Each row in the annotation table results in n rows in the annotation table, each holding the concept identifier (ConceptID) and a concept label (ConceptLabel) from the ontology and a level denoting the semantic distance (the super classes of the annotation concepts are also stored)

First, we created a single table for each source column that had to be annotated. The results are annotation tables that contain semantic representations of the content of the source columns. The precondition is that each table contained a unique primary key, which held true in practice. For each row of the given column, we created n rows in the annotation table that contained the semantic interpretations. These annotations are the specific concept identifiers in the selected ontology. The records are linked via the primary unique key. In addition to storing direct annotations, we also stored the respective super classes (derived from the “is a” hierarchy from the ontology) to enable a performance analysis. Figure 2 shows the designed structure, and Table 1 shows a sample data set.

**Table 1:**
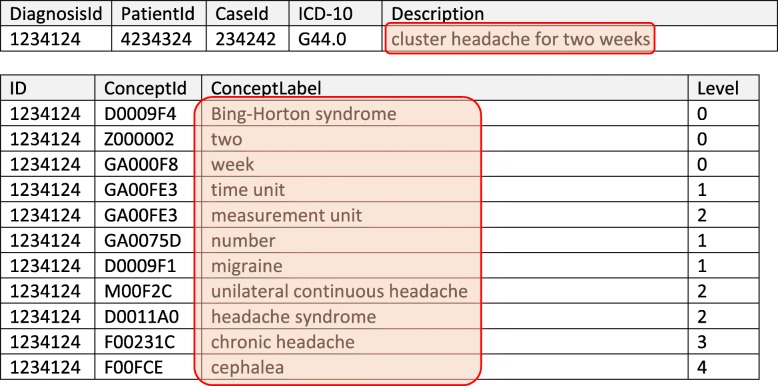
Sample of the resulting data structure. The upper table represents a diagnosis table that has a primary unique key (DiagnosisId), a patient and case key, an ICD code and a description of the diagnosis. The diagnosis “cluster headache for two weeks” is annotated with “D0009F4 Bing-Horton syndrome Z000002 two GA000F8 week” and subsequently stored in the annotation table (lower table). The semantic distance is “0” here because the concepts directly represent the narrative description of the diagnosis. Additionally, the parents of all concepts found are stored in the annotation table with the same diagnosis id. Therefore, “cephalea” is a parent of the 4th degree of “cluster headache”. The parent concepts are retrieved with a function call from the terminology server that returns the taxonomy of a given concept. The tables are linked with the relation “DiagnosisId ➔ Id”

The approach of using a generic table with metainformation for the description of the “source table” was discarded in favor of performance or respective costs. The annotation table schema is fairly simple with four columns (one for the key, one for the concept identifier, one for the concept label and one for the semantic distance (level)), and thus, modern RDBMS are expected to handle such tables extremely well. As an additional benefit of this solution, semantic data models can be generated from analysis of the annotations, especially if the source column contains keyword data [25].

The annotation of free text is accomplished by integrating a CTS2 (common terminology services)-compatible terminology server [26]. The terminology server used here includes a complete NLP pipeline based on Gate [27] and Jape in addition to numerous supporting algorithms, such as an extensive, discipline-specific list of abbreviations, collocation-based disambiguation, a typing error corrector, which can break up compounds and correct them step by step, and a function for German language-optimized word stemming (for further information, see Additional file 1: Addendum 1).

### Ontology-SQL syntax

We developed an extension to standard SQL that enables the use of free-text and semantic relations within such a query. Expressions formulated through this extension can be transformed into standard SQL syntax using a preprocessor. The resulting query contains only standard SQL and thus can be directly passed on to the database server engine.

A basic O-SQL expression consists of a free-text part that is surrounded by square brackets and followed by a table and column name in which the free-text should be searched for. The latter is surrounded by round brackets. So the simpliest expression looks like this:

[free-text](tablename.columnname)

Furthermore, the table and column name can be a comma separated list. Since the free-text is mapped to the ontology, a semantic role (or in short: relation) can be given that will be applied to the expression. That relation is a keyword written before the O-SQL expression:

relation[free-text](tablename.columnname)

The default value for the relation is “isA”, which would query all concepts subsumed under the concept described in the free text.

The generic relation “context” can be further specified by a modifier to extent the standard relations like “is a” and “part of” to relations like “has indication”. This context modifier is a free-text written in curly braces between the relation and the free-text query:

context{modifier}[free-text](tablename.columnname)

The approach of a context modifier was chosen to allow a generic, ontology independent syntax of O-SQL expressions.

**Fig. 3:**
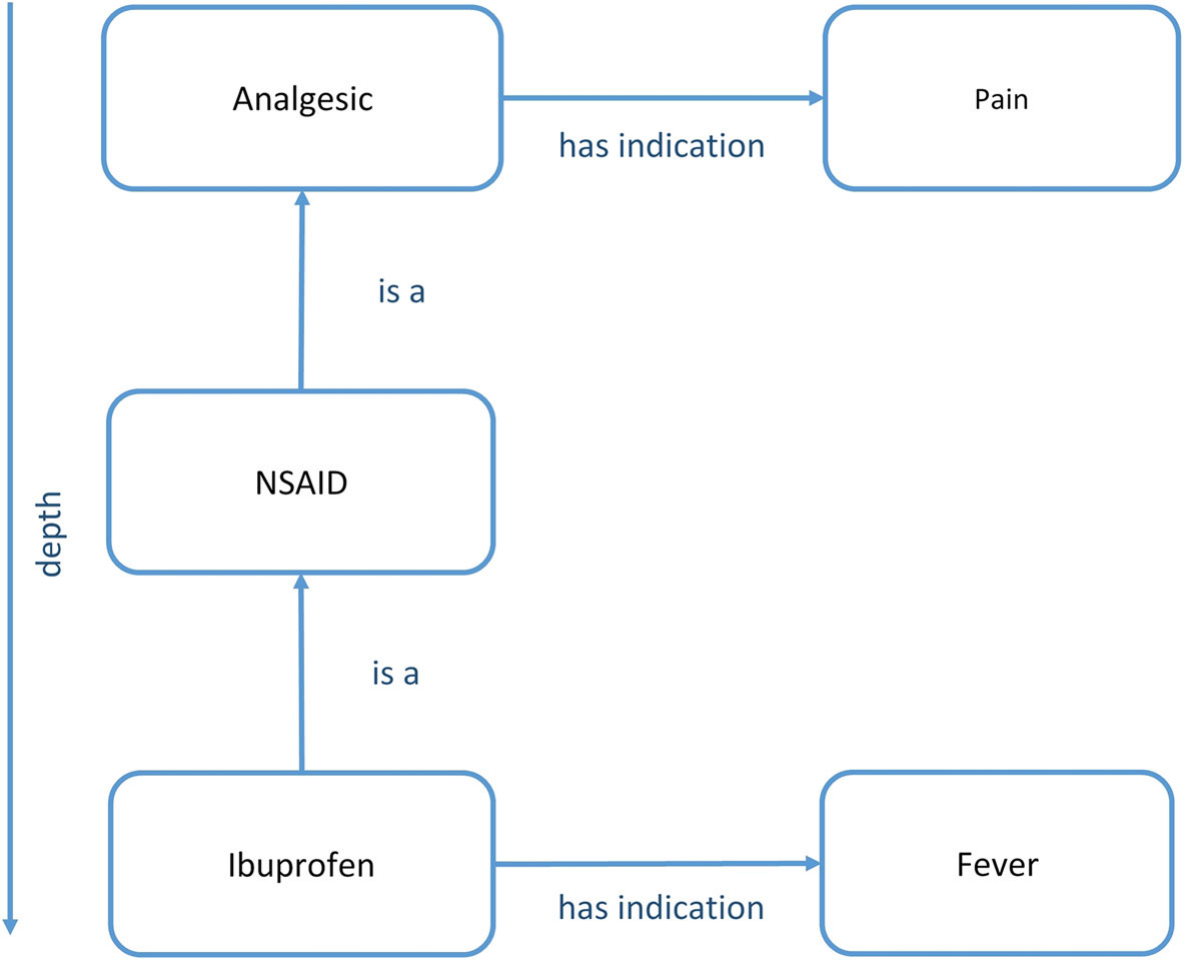
Inheritance via depth parameter: in this sample, indications of the concept “Analgesic” subsum indications of “Ibuprofen”; thus, a query for indications of “Analgesic” will find “Pain” and “Fever” if the depth is set to ≥2

As shown in Fig. 3, the attributes of the semantic roles can be passed on through the isA-hierarchy, which allows inheritance of these attributes up to a specific depth. The inheritance depth is specified by a number separated from the context modifier by a colon. Finally, a leading prefix can be added to include the “is a” relation to the given relation.

Consequently, the complete syntax for O-SQL expressions is as follows:

[prefix][relation][{modifier}][:depth][[query]](table.column,…)

Its elements and their values are further illustrated in Additional file 1: Addendum Table S1.

When designing the syntax, we attempted not to focus on a functional characteristics but rather decided to maintain the narrative character of a “query”. A free-text formulation of the actual query offers enormous advantages as the knowledge base is seamlessly integrated and implementation details of the underlying terminology and ontology are hidden. Liebermann et al. demonstrated early on that SQL-based queries of annotated databases can provide very high recall values, but the respective queries required extensive knowledge of the underlying ontology and manual research on the ontology concepts [28]. Since the terminology server provides an NLP engine, the query is tolerant to typing errors, and even complex medical concepts requiring post-coordination can be used.

### Conversion

An efficient conversion of O-SQL into standard SQL is crucial, as this step mainly affects the runtime of given queries. First, O-SQL expressions are extracted via regular expressions and are then converted into SQL subqueries.

We decided to use the IN operator for subqueries because the source table holds a primary unique key and the subclause can be a simple enumeration of the found annotation rows. Modern RDBMS use a so-called “Clustered Index Scan” to process such queries efficiently.

For each O-SQL statement, we created a subclause by first mapping the free-text part of the O-SQL expression onto the terminology and then querying the annotation table for the found concept identifiers. From the results of these queries, the values of the column containing the key were used to build the subclause. Then, the O-SQL expression was replaced by that subclause. All other parts of the SQL query remained untouched. Therefore, all logical operators and language components – especially parentheses and the use of the logical operator NOT – function as usual so that structured discrete information can be directly linked to semantic information.

Given an O-SQL query such as the following:

SELECT * FROM table_name WHERE <O-SQL expression>

We transform the free-text part of the O-SQL expression with the help of the terminology server to form concept identifiers and look them up in the annotation table (see Table 1):

SELECT ID FROM annotation_table WHERE conceptID = <concept identifier>

We then join all the results together and replace the O-SQL expression with an “IN” subquery:

SELECT * FROM table_name WHERE ID in (…)

If a query contains multiple O-SQL expressions, each expression is converted separately. Therefore, one can use all standard SQL operators to combine O-SQL expressions. For an example, see Additional file 1: Addendum 2.

**Fig. 4:**
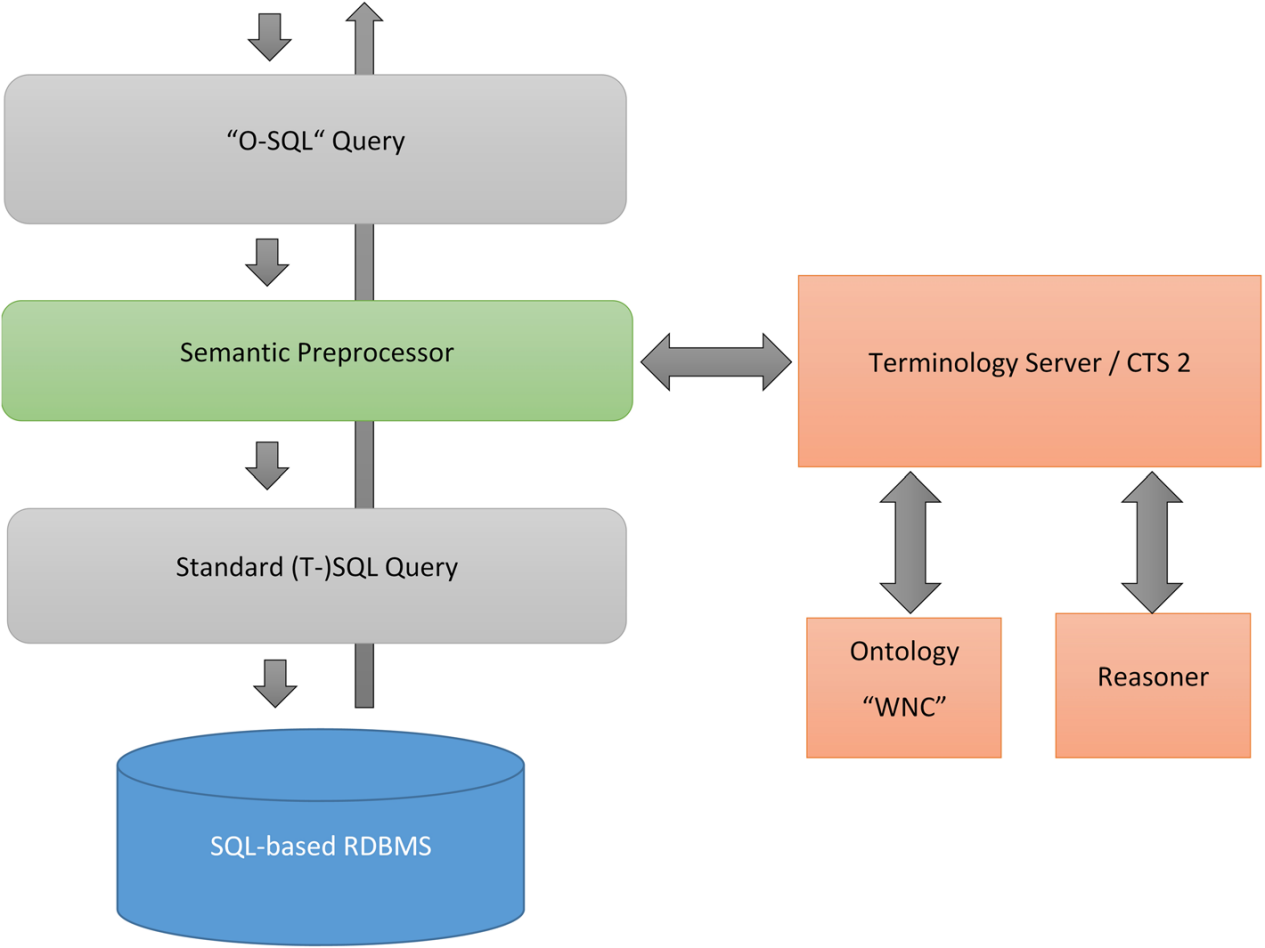
Schematic overview of transforming an “O-SQL” query into a standard SQL query using a standardized terminology server

Figure 4 illustrates the entire pipeline via which semantic queries can directly be integrated into standard SQL. The pipeline also provides feedback on the terminology concepts actually used to avoid undetected errors. Such errors can happen if an abbreviation is not known and is therefore misinterpreted.

## Results

First, we measured the accuracy of the annotation process to evaluate the results gathered from O-SQL queries. We then analyzed published knowledge-based queries from various publications to show whether and how our approach was applicable and able to simplify them. Finally, we examined the data with specific queries and measured the results with respect to precision and recall. For all statistical calculations, we used MedCalc® [29].

### Annotation accuracy

The annotation algorithm of the terminology server could recognize abbreviations and had a spell check function optimized for the German language and a module for disambiguation of semantic interpretations. In particular, the spell check function was urgently needed since German allows the construction of so-called “compound nouns” and since many of these compound nouns had been shortened into their subwords and then reconstructed again. A typical example was the German term for pregnancy advisory, which is “Schwangerenberatung”; this term was often written as “Schw.brtg” in many variants.

We did not set up a standardized procedure that included a manual annotation and an inter-annotator agreement to calculate precision and recall for two reasons: I) we were mainly interested in whether the annotation was correct and not whether the annotation was optimal (focus on precision and not on recall) and II) the architecture is independent of the terminology server and uses a standard interface for the integration. Thus, any CTS compliant terminology server can be easily used.

We manually analyzed the automated annotation from 10% (*N* = 187) of the cards, namely, the section that contained the postmortem diagnoses. We found 423 diagnoses, 304 of which were unique. Each annotation was classified as “completely correct”, “partly correct” or “incorrect”. The category “partly correct” contained items that could not be mapped better due to missing precoordinated concepts in the ontology, and thus, the expert partly disagreed with the interpretation (e.g., “aspiration of infected amniotic fluid” was annotated as “aspiration of amniotic fluid” and “infection”). The category “incorrect” contained all items that were incorrect. In total, we found that 301 of the 304 (99.0%) items were correctly mapped or could not be mapped better. Twenty-three of these items were in the category “partly correct” and could be fixed by adding new (precoordinated) concepts to the terminology. The remaining three were “incorrect”.

Furthermore, annotation quality was evaluated using a single concept from the terminology, namely, the anatomy concept “tentorium”. We analyzed all 1868 cases with a total of 9080 postmortem descriptions and noted whether the concept “tentorium” was present. We found 50 different spellings in 77 cases. For all of these cases, we verified whether the mapping algorithm used the correct concept. From these comparisons, we found a sensitivity of 97.62% (CI 95%: 91.66–99.71%) and a specificity of 100.00% (CI 95%: 98.87–100.00%) for the automated annotation.

### Published queries

We analyzed published queries and examined whether we were able to express them in O-SQL and whether that expression was more compact and easier to create and understand. The following section demonstrates that these expectations held true for all examined samples.

In Lieberman et al., the request for patients with “coronary artery disease” resulted in the following partial expression [28]:

concept_id in (

   select concept_id from snomed_map

   where snmd_cncpt = 8957000 or snmd_cncpt in (

   select snmd_cncpt1 from snmd_relationship

   connect by snmd_cncpt2 = prior snmd_cncpt1 and

   relationship_type = 116680003

   start with snmd_cncpt2 = 8957000 and

   relationship_type = 116680003)

)

Its complexity is basically derived from the need to use nested subqueries to represent relationships within the ontology. The IDs are from SNOMED CT:

8957000 = Coronary artery disease (disorder)

116680003 = Is a (attribute)

This query can be represented in O-SQL by the following compact expression using a common abbreviation:

[chd](diagnosis_column)

The next sample shows that the complexity of SPARQL can also be greatly reduced. In [30], the filter criterion “Find all patients having a side effect of Prandin after administration” is defined. Pathak et al. transformed this criterion into the following SPARQL query (abbreviated):

SELECT DISTINCT ?MCLSS_KEY {

 { SERVICE <http://www4.wiwiss.fu-berlin.de/sider/sparql>

 { SELECT ?mySideEffect ?mySideEffectLabel WHERE {

 ?x rdf:type sider:drugs ;

 rdfs:label "Prandin" ;

 sider:sideEffect ?mySideEffect .

 ?mySideEffect rdfs:label ?mySideEffectLabel .

 }}}

 { SELECT DISTINCT ?rxnormCode WHERE {

   SERVICE <http://link.informatics.stonybrook.edu/sparql/> {

 ?rxAUIUrl rxnorm:hasRXCUI ?rxCUIUrl ;

 rdfs:label ?rxnormLabel .

 ?rxCUIUrl rxnorm:RXCUI ?rxnormCode .

 FILTER(regex(str(?rxnormLabel), "Prandin", "i")) .

 }}}

 { SELECT DISTINCT ?MCLSS_KEY WHERE {

   SERVICE <http://edison.mayo.edu/lss1p#> {

 ?icd9Url semr:dx_code ?icd9Code ;

 semr:dx_abbrev_desc ?diagnosis .

 FILTER(regex(str(?diagnosis),

 str(?mySideEffectLabel), "i")) .

 ?patientUrl semr:whkey ?MCLSS_KEY ;

 semr:diagnosis ?diagnosisCode ;

 semr:concept_id ?rxnormCode .

 FILTER(regex(str(?icd9Code),

str(?diagnosisCode), "i")) .

}}}}

This query requires a deep understanding of SPARQL and the structure of external knowledge bases. In addition, this query requires that local diagnoses are encoded in ICD-9, as the medication database uses this classification to structure information on “side effects”.

In contrast, the query can be drastically reduced to the following form using our approach:

select * from tableMed, tableDiag where

   tableMed.CID = tableDiag.CID and

   +partOf[Prandin](tableMed.Drug) and

hasContext{side effect} [repaglinide](tableDiag.Diag)

The first partial expression searches the column “Drug” in the table “tableMed” for all occurrences of “Prandin” itself (note the “+”) and for all concepts containing “Prandin”. In doing so, the agents of Prandin are also found, and possible generic drugs are included. The second partial expression simply scans the ontology for a “side effect” of the agent and uses these results to search the column “Diag” in the table “tableDiag”. Here, it must be ensured that specifications are also found in each case. Therefore, Prandin has “hypoglycemia” as a side effect and with the assistance of the ontology, the query will also identify patients in which “hyperinsulinism” is recorded because “hyperinsulinism” is a form of “hypoglycemia”.

The last example was published by Leroux and Lefort, who queried “anti-diabetic drugs, such as Metformin,” therefore defining the following request (abbreviated) [31]:

SELECT count (distinct ?subject) as ?count ?mp_med WHERE {

SERVICE <http://wifo5-04.informatik.uni-mannheim.de/drugbank/sparql>

{

 ?s drugbank:genericName "Metformin" .

 ?s drugbank:drugCategory ?category .

 ?drug drugbank:drugCategory ?category .

}

{ SELECT distinct ?drug ?med ?subject ?mp_med WHERE {

 GRAPH <http://localhost/dataset/aibl/lcdc/clinical> {

 ?obs a lcdcobs:Observation .

 ?obs cm:medicinalProduct ?cm_mp .

 ?cm_mp skos:exactMatch ?drug .

 ?cm_mp amt:synonym ?mp_med .

 ?obs lcdccore:subject ?subject .

}}}}

Additionally, in this case, the query can be drastically reduced when using O-SQL:

select * from tableMed where hasContext{indication}:5[diabetes](Drug)

The indications for all children are determined recursively from “diabetes” up to the specified depth of “5” (semantic distance).

### Specific queries

We used three typical neonatal complications to verify the querying capabilities: (I) all of the of the index cards were manually reviewed to see whether the patient had a “vitium cordis”, (II) 25% of the index cards were manually reviewed for the presence of a sign of “respiratory disorder”, and (III) we used the yellow highlighting on the index cards to carry out a full scope examination of all 1868 index cards for “lethal malformation”. We then created corresponding O-SQL queries to look up these groups, compared the results and calculated standard statistics (see Table 2).

**Table 2:** Statistical results of semantic queries of different complexity (complexity is represented by number of query concepts). All statistics are calculated with MedCalc® [29]. Raw data can be found in Additional file 1: Table S2 in the addendum

Query	“Vitium cordis”	“Respiration disorder”	“Lethal malformation”
Number of query concepts	1	5	16
Sample size (Percentage of all cards)	1868 (100%)	467 (25%)	1868 (100%)
Sensitivity	93.22% (CI 95%: 89.22–96.08%)	97.76% (CI 95%: 95.79–98.97%)	92.13% (CI 95%: 89.48–94.29%)
Specificity	99.94% (CI 95%: 99.66–100.00%)	95.45% (CI 95%: 87.29–99.05%)	96.30% (CI 95%: 95.16–97.24%)
Positive predictive value	99.55% (CI 95%: 96.87–99.94%)	99.24% (CI 95%: 97.75–99.75%)	90.57% (CI 95%: 87.75–92.92%)
Disease prevalence	12.57% (CI 95%: 11.10–14.15%)	85.90% (CI 95%: 82.41–88.92%)	27.80% (CI 95%: 25.78–29.89%)
F score	0.96	0.98	0.91

## Discussion

We developed an approach that enables free-text queries stored in standard SQL-based RDBMS. Thus, we extended standard SQL syntax and created an architecture that allows us to integrate a terminology server into existing databases.

The usefulness of historical data is becoming clearer due to the tremendous progress in the development and availability of medical terminologies and ontologies and in the field of NLP [32]. However, when comparing the specific findings of queries in this work with the results of Epstein [17], it is striking that in certain areas, the ontology first had to be extended to enable the use of historical text. This extension was required for the recognition or annotation of drugs, as trade names that are no longer in use had been employed.

We overcame the disadvantage of Zheng’s approach [18], which needed an already annotated database, by integrating a terminology server into the analysis pipeline. In addition, the user is not required to have any knowledge about how the terminology works since the data annotation and free-text annotation of the query expressions use the same NLP-based terminology server.

The annotation results outperformed the approaches presented by Allones et al. [5] and Shah et al. [7] and compared very well to the ones presented by Topfer et al. [6], who obtained slightly better results, and Elkin et al. [4], whose results were slightly inferior to ours. This comparison of final metrics shows that the employed NLP pipeline and annotation algorithm deliver state-of-the-art results and provide an objective evaluation of the query results. Since the results come from a relatively small set of data, they can only be generalised if the terminology is complete and homogeneous, which needs to be validated. Maintaining terminology and ontology was not a specific topic of our research, as we used a standardized terminology server that can provide different terminologies and is easily interchangeable.

The slight loss of sensitivity when querying for heart defects was traced back to 16 cases that were false negatives. The reasons for these false negatives were unrecognized abbreviations, missing precoordinated terminology concepts, missing ontology links and in one case, selection of an incorrect term-to-concept combination by the annotation algorithm. However, the results compare very well to those of Pakhomov et al. [33].

The imperfect specificity for respiration disorders can be explained by misinterpreted cases of “intrauterine hypoxia” and the reduced sensitivity was mainly due to cases with a “hyaline membrane” that were not correctly classified.

The decreased sensitivity in the cases of malformation was mainly caused by the inconsistent gold standard. The original marking was performed under the assumption that the malformation ultimately led to death, since this malformation influenced the original judgment of avoidability. This situation becomes particularly clear in the case of congenital tumors. Systematic yellow marking in the case of teratomas showed that these tumors had been generally recognized as lethal malformations, whereas some neuroblastomas were not included in this category. The overall quality was still excellent, as over 90% of the individuals were correctly classified with this rather difficult medical definition.

When comparing the complexity of SPARQL queries to O-SQL queries, it became clear that a considerable advantage was not only the ability to use free text but also the commonly available knowledge of the SQL syntax. We assume that committed physicians with existing knowledge of SQL can be trained to use O-SQL without issue. In particular, clinicians’ calls for “secondary use” have caused big software companies to open up their databases to clients. The use of routine systems, however, bears certain limits, as the data must not be changed, and the stability of the database models is not provided per se. The first limit is completely circumvented by the framework presented here, as all annotations are stored in their own tables and the original tables remain untouched. Changes in the database schema with respect to the data model can be represented quite easily by re-generating the annotations. Also, when evaluating the differences, it is important to note that SPARQL is designed to query RDF data and SQL is designed to query relational data. Thus, the advantages of both languages directly reflect the data models on which they work [34].

Physician training on the use of O-SQL expressions must be conducted in two steps: first, learning the actual syntax, which was rather unproblematic, and second, learning how to use the “mechanics” of an ontology. The latter could be addressed by providing a detailed demonstration with examples, which could later be adopted, of what an ontology is and how it can be used. Without such a demonstration, physicians tend to use rather simple queries, which do not exploit the full power of the ontology.

## Conclusions and future work

Many of the difficulties in using ontologies for the semantic analysis of free text that were described in the introduction of this paper were overcome by the approach presented here. The overall concept of integrating an NLP-based terminology server into SQL syntax has been proven to be extremely viable.

In particular, the high complexity and diversity of the German language were processed at a quality that meets the requirements of medicine. The terminology server used here is multilingual, so the O-SQL syntax simply had to be extended to specify the language used. With this approach, it is now possible to compare databases on an international level, and country-specific annotations with only one uniform query language, such as German or English, could be implemented. A first application, in which neotalogic epicrises were analysed with regard to avoidable infant mortality, has already proved successful [35].

The ontology-SQL syntax will be extended to allow for nested expressions. For this purpose, the extent to which these expressions are relevant and whether they cannot perhaps be represented by concatenated expressions will have to be evaluated. One example would be a query for “patients showing symptoms of diseases that can be treated with specific agents”.

From a medical point of view, queries for rare diseases should be investigated. Here, selection will have to be carried out via O-SQL and will be followed by concrete case-by-case examinations.

In addition, the ontologies themselves (especially the standard ontologies) will become increasingly extensive as more and more “omics data” are represented. Genetic information that was unknown at the time of data collection can thus be considered in queries using the approach presented here. Deriving quality factors from historic data is of growing interest because it enables a comparison of historic procedures and treatments to the current medical state-of-the-art.

The approach presented here is independent of a specific ontology, on the contrary it allows access to any number of ontologies. However, also controlled vocabularies can be made applicable through this system. Therefore, physicians can access and explore data with specially developed ontologies that go beyond the spectrum of standard ontologies. This removes one of the typical limitations of standard ontologies, which cover a broad spectrum of knowledge but usually have a limited depth. This should significantly increase the acceptance of the system. The fact that routine data from many sources – as long as it is stored in SQL based databases - can be immediately used at an ontology-driven level without further transformation or integration demonstrates that the presented work makes an important contribution to translational research of routine data.

## Additional file

Additional file 1: **Addendum 1.** Detailed description on the specific used terminology and ontology. Explaines some of the advanced features possible. **Addendum 2.** Further details on O-SQL expressions, namely on the rewriting process. Also contains a structured documentation of the parts of an O-SQL expression. (DOCX 57 kb)

## Abbreviations

CI: Confidence interval
CTS: Common terminology services
GMDS: Deutsche Gesellschaft für Medizinische Informatik, Biometrie und Epidemiologie
ICD: International classification of diseases
NLP: Natural language processing
OCR: Optical character recognition
OLAP: Online analytical processing
OWL: Web Ontology Language
RDBMS: Relational database management system
RDF: Resource Description Framework
SEP: Structure Entity Part
SPARQL: SPARQL Protocol and RDF Query Language
SQL: Structured Query Language

## Footnotes

1 Infant mortality rate (IMR) = the number of deaths of children under 1 year of age per 1000 live births
