# Supplementary material for: Integrating terminologies into standard SQL: a new approach for research on routine data
Source: J Biomed Semantics. 2019 Apr 24;10:7. doi: 10.1186/s13326-019-0199-z (PMC6480592; doi:10.1186/s13326-019-0199-z)
Supplement: Supplementary file 1 — Addendum 1. Detailed description on the specific used terminology and ontology. Explaines some of the advanced features possible. Addendum 2. Further details on O-SQL expressions, namely on the rewriting process. Also contains a structured documentation of the parts of an O-SQL expression. (DOCX 57 kb) [file 13326_2019_199_MOESM1_ESM.docx]

**ADDENDUM 1**

The origins of the here used mapping algorithm are going back to a paper by Wingert, who in 1987 described how annotation of ICD classifiation texts can be established using SNOMED concepts [30]. However, as this approach was improved by modern NLP elements, we refer to and compare our results with results from mainly recently published papers, ensuring state of the art comparisons. The mapping basically matches a number of word stems from the input text to the word stems of the terminology concept. This process is optimized towards minimization of the number of such concepts. This way, it is ensured, that the concepts with the highest information density are used. Unlike stated by Allones et al [6] who said, that there exist no mapping algorithms that are capable of using synonyms but the here used algorithm uses indeed synonyms. This is a crucial must, as it allows for compact modelling of the terminology:

Let concept C_1_ be labeled by term T_1_ (“heart infarction”) which consists of the sub-terms ST_1_ (“heart”) and ST_2_ (“infarction”)

Let concept C_2_ be labeled by the terms ST_1_ (“heart”) and a synonymous term ST_3_ (“cardiac”)

Then concept C_1_ has to be found by term ST_3_ plus ST_2_ (“cardiac infarction”), even so, it’s not labeled with these terms

Thus, the mapping algorithm first matches all input terms to “atomic” terminology concepts to retrieve synonyms, to then do the actual mapping with the above-described optimization. A very important addendum for languages with compounding is that since the algorithm works on base of word stems, it also works perfectly for compound nouns.

For disambiguation, the NLP engine combines heuristic approaches that use machine-learning algorithms with the well-established method of word collocations but also makes use of the ontology itself. If a word, abbrebivation or phrase has multiple meanings, the ontology is used for the calculation of semantic distances, which helps in guessing the correct meaning.

For the German-speaking area, there is at present only one terminology that both covers the medicine to the here required extent and is expressible in description logic. This terminology is the Wingert nomenclature (WNC) of the Friedrich Wingert Foundation. Its roots lie in SNOMED 2, although over the last decades and in analogy to SNOMED CT, its structure has been formalized and the Wingert nomenclature has been transformed and can now be expressed in dialects of description logic, like AL (attributive language) and EL (existential language) [31].

In contrast to SNOMED, the WNC does not contain generic concepts that can be used as placeholders. Typically, such concepts are used in classifications to allow coding of items into categories, which are “not otherwise specified”. The disadvantage of such concepts is shown in [32].

The particularity of the WNC is that all semantic roles are represented using a generic approach. The semantic bottom role is simply designated as "hasContext" and further defined through a parameter. This parameter in turn is an element of the terminology itself.

The representation of "has_Topography" or respectively "has_FindingSite" is consequently carried out using the expression "hasContext{Topography}". The general notation is "hasContext{parameter}". The advantage of this approach is that the roles themselves are represented by the ontology so that they can also be semantically "understood". This benefit becomes clear in the following example:

Let X1 ≡ ∃hasContext{R1}.Y1 and

X2 ≡ ∃hasContext{R2}.Y2 and

R1 c R2, then follows

X2 c ∃hasContext{R1}.Y2

Transformation of that approach into standard notation and proof for the expressibility in AL and EL was done using Protegé [33] and public available reasoners (ELK, PELLET) [34, 35].

The use of certain perspectives in the maintenance of an ontology inevitably leads to contradictory models. This holds true in a general sense for aspects of the TCM (Traditional Chinese Medicine) as opposed to orthodox western medicine, which often makes subsumptions that do not reflect medical reality. These aspects can be represented in the WNC by using so-called "views". In so doing, we basically parameterize the modeling of the ontology. The approach of working with "views" is especially used for the implementation of negations, although it’s only used to suppress inheritance along the “is-a” relation.

The modeling of the partOf relation is associated with huge problems, as outlined by Schulz and Hahn [36]. The propagated "SEP (Structure Entity Part) approach" goes already back to the 80ies and was introduced and discarded by Young et al. [37]. Although this approach had first been chosen for SNOMED CT, today alternatives are discussed. In the WNC, the partOf relation was not modeled transitively but especially used for the representation of anatomical relationships. Conceptual relations like, for instance, "drug c ∃hasPart.active_agent" or "bone_fracture c ∃hasPart.bone ∩ fracture" were modeled in the sense of conceptual relations using the generic “hasContext”-approach, leading, for example, to terms like "drug c ∃hasContext{concept}.active_agent". Thus this type of partonomic relation describes intrinsic parts.

The here used terminology server is CTS2 compliant [38], which is a standard that has emerged from Open Terminology Services [39]. The use of a standardized tool for the integration of the knowlegde allows for an uncomplicated exchange of the implementation and enables a flexible use of different ontologies. Among others, the backbone of the NCBO bioportal, which contains almost 600 terminologies and ontologies, is based on CTS2 [40]. Therefore, all these ontologies can, in principle, be used when working with our approach. The CTS can be connected to a reasoner, which evaluates DL expressions in the chosen dialect.

**ADDENDUM 2**

In the following, the conversion is explained on a simple example. So, it shall be queried for patients of a certain age with an allergy. For this purpose, the O-SQL query is formulated as follows:

select p.id as patient_id from patients p, allergies a where p.age > 65 and p.id = a.patient_id and **[penicillin](a.allergy)**

The tables "patients" and "allergies" are connected via the relationship "p.id = a.patient_id" and the free-text allergies are documented in the table “allergies” and column “allergy”. The preprocessor then extracts the expression "[penicillin](a.allergy)", retrieves the concept-id for "penicillin" with the assistance of the integrated terminology server and resolves the references by means of the annotation tables. Thus, the resulting standard SQL is:

select p.id as patient_id from patients p, allergies a where p.age > 65 and p.id = a.patient_id and **a.id in (23, 33, 45)**

By means of this statement, now all patients are selected who are older than 65 years and for whom an allergy to "penicillin" has been recorded. This query includes, on the one hand, patients suffering from an allergy to one specific penicillin like, for example, Oxacillin or Amoxicillin, and on the other hand, it excludes those patients for whom such an allergy had been ruled out in the free-text documentation (e.g. "no allergy against pencillin")

**TABLES**

| Element | Values | Sample |
| --- | --- | --- |
| Prefix | If the prefix is set to “+” the relation “isA” will be added to the given relation | “+partOf[bone]” equals to “partOf[bone] or isA[bone]” |
| Relation | - isA (default) - parentOf / isA - partOf / hasPart - isContextedBy / hasContext - context (equals to isContextedBy and hasContext) - siblingOf | “isA[inflammation]” will also find “phlegmon” and so on |
| Context | A free-text parameter further specifying the “context” relation | “hasIndication” is expressed by “context{Indication}” |
| Depth | Number specifying the inheritance depth; see explanation in the main text and in Figure 3 |  |
| Query | The free-text query; can be a single keyword or a complete phrase including negations | “chd”, “diabetes without complication” |
| Table.Column | Name of the table and column to search in; can be a comma-separated list |  |

Table S1 Possible values of the elements of an O-SQL expression.

| Query | TP | FP | TN | FN |
| --- | --- | --- | --- | --- |
| Vitium cordis | 220 | 1 | 1641 | 16 |
| Respiration disorder | 393 | 3 | 63 | 9 |
| Lethal malformation | 480 | 50 | 1303 | 41 |

Table S2 Raw data used for statistics calculation. TP = true positve; FP = false positive; TN = true negative; FN = false negative

**ADDENDUM REFERENCES**

[6] Allones JL Martinez D, Taboada M, Automated mapping of clinical terms into SNOMED-CT. An application to codify procedures in pathology. [J Med Syst.](https://www.ncbi.nlm.nih.gov/pubmed/?term=Automated+mapping+of+clinical+terms+into+SNOMED-CT.+An+application+to+codify+procedures+in+pathology) 2014 Oct;38(10):134. doi: 10.1007/s10916-014-0134-x. Epub 2014 Sep 2.

[30] Wingert F, Automated indexing of SNOMED statements into ICD., Methods Inf Med 1987 26: 93-98

[31] Baader et al, The description Logik Handbook, Cambridge Press (2003)

[32] Ruch P, Gobeill J, Lovis C, Geissbuehler A, Automatic medical encoding with SNOMED categories, BMC Medical Informatics and Decision Making 2008, 8(Suppl 1):S6

[33] Musen, M.A. The Protégé project: A look back and a look forward. AI Matters. Association of Computing Machinery Specific Interest Group in Artificial Intelligence, 1(4), June 2015. DOI: 10.1145/2557001.25757003.

[34] Kazakov Y, Kroetzsch M, Simancík F, Concurrent Classification of EL Ontologies. Technical report. University of Oxford 2011

[35] Evren Sirin a , Bijan Parsia a , Bernardo Cuenca Grau a,b , Aditya Kalyanpur a , Yarden Katz, Pellet: A Practical OWL-DL Reasoner, Web Semantics: Science, Services and Agents on the World Wide Web, Vol. 5, Issue 2, (2007):51-53

[36] Schulz S, Hahn U, Part-whole representation and reasoning in formal biomedical ontologies, Artificial Intelligence in Medicine (2005) 34, 179—200

[37] Yang Y, Patil R. KOLA: a knowledge organization language. In: Kingsland III LC, editor. SCAMC’89 — Proceedings of the 13th annual symposium on computer applications in medical care. New York, NY: IEEE Computer Society Press; (1989). p. 71—5.

[38] Common Terminology Services 2 http://www.omg.org/spec/CTS2/ (Accessed: 06.11.2016)

[39] Solbrig HR, Armbrust DC, Chute CG, The Open Terminology Services (OTS) project., AMIA Annu Symp Proc. (2003):1011.

[40] NCBO Bioportal, http://bioportal.bioontology.org/ (Accessed: 07.11.2016)
